# Stabilisation of beta and gamma oscillation frequency in the mammalian olfactory bulb

**DOI:** 10.1186/1471-2202-12-S1-P362

**Published:** 2011-07-18

**Authors:** Nicolas Fourcaud-Trocmé, Emmanuelle Courtiol, Nathalie Buonviso, Thomas Voegtlin

**Affiliations:** 1 INSERM U1028; CNRS UMR5292; Lyon Neuroscience Research Center, Olfaction: from coding to memory Team, Lyon, F-69000, France; 2 University Lyon 1, Lyon, F-69000, France; 3 Equipe Cortex, INRIA Lorraine; Vandoeuvre-les-Nancy, France

## 

The dynamics of the mammalian olfactory bulb (OB) is characterized by local field potential (LFP) oscillations either slow, in the theta range (2-10Hz, tightly linked to the respiratory rhythm), or fast, in the beta (15-30Hz) or gamma (40-90Hz) range. These fast oscillations are known to be modulated by odorant features [1] and animal experience or state [2][3], but both their mechanisms and implication in coding are still not well understood. In this study, we used a double canulation protocol to impose artificial breathing rhythms to anesthetized rats while recording the LFP in the OB. We observed that despite the changes in the input air flow parameters (frequency or flow rate), the main characteristics of fast oscillations (duration, frequency or amplitude) were merely constant. We thus made the hypothesis that fast beta and gamma oscillations dynamics are entirely determined by the OB network properties and that external stimulation was only able put the network in a state which permits the generation of one or the other oscillations (they are never present simultaneously).

To test this hypothesis, we studied a simplified OB model based on previous modeling studies. In particular it includes resonant mitral cells [4] and graded synaptic inhibition [5]. Detailed analysis and numerical simulations of the model showed that two oscillatory dynamical regime can be sustained.

First, a low noise regime where at each cycle, a subset of mitral cells are tightly synchronized and yield a saturation of the graded inhibition, during which mitral cell discharge is prevented. Because of this saturation, the network frequency is governed by the dynamics of the graded inhibition decay. Interestingly, this dynamics is not very sensitive to the number of mitral cells that lead to the saturation. Consequently, there is a wide range of mitral cell firing rate during which the network frequency is stable. Using standard parameters in our model, we found an oscillation frequency stable between 25 and 30Hz while the average mitral cell firing rate increased from 5Hz to 25Hz.

Second, a high noise regime can be observed where the mitral cell discharge is much less regular. In this case, the network oscillations are of lower amplitude compared to the low noise regime and thus the graded inhibition is never saturated by fluctuations around its mean. The network can then be well described by a mean field approach (as described in [ 6]) and is close to a previous model of in vitro gamma oscillations [4]. With our model parameters, oscillations in this regime have higher frequency, typically in the gamma range (50Hz-90Hz) but their frequency is highly sensitive to the level of input excitation to the mitral cells. This discrepancy with our experimental result could be relieved by the in vitro experimental observation that mitral cell stimulus excitatory input is all or none [7]. Indeed this could provide a stable external input to the network and thus also stabilize the gamma frequency.

Finally, we have shown that our model can account for the two stable oscillatory regimes observed in the OB in vivo. However our model does not take into account the spatial activation of the OB and the study of how spatially segregated oscillatory generators interact and potentially synchronize is an ongoing work.
